# Mendelian randomisation in ophthalmology: An overview and practical guide for clinicians

**DOI:** 10.1177/11206721261461915

**Published:** 2026-06-29

**Authors:** Kelsey V. Stuart, Alasdair N. Warwick, Claudia Liang Peng, Skanda Rajasundaram, Anthony P. Khawaja

**Affiliations:** NIHR Biomedical Research Centre, Moorfields Eye Hospital NHS Foundation Trust and 4919 UCL Institute of Ophthalmology, London, UK

**Keywords:** Mendelian randomisation, ophthalmology, review

## Abstract

Mendelian randomisation (MR) is an epidemiological approach that leverages natural genetic variation to strengthen causal inference. When applied rigorously and interpreted within a broader framework of evidence, it offers a powerful tool to advance understanding and inform clinical translation. However, misuse or misinterpretation of the technique is increasingly common and risks introducing spurious or implausible findings into the scientific literature. Violation of core underlying assumptions and lack of clear gene-environment equivalence often negates meaningful clinical translation, and overreliance on MR results without triangulation with other lines of evidence may lead to misleading causal claims. As the use of MR continues to expand within ophthalmic research, it is increasingly important for clinicians and researchers to be equipped to critically appraise and interpret such studies.

Here we outline the principles underpinning MR, including its core assumptions and methodological evolution. We summarise its application across major ophthalmic conditions, highlighting its role in validating risk factors, clarifying causal pathways, and identifying potential therapeutic targets. We then examine key limitations and common pitfalls of the approach, before providing practical guidance for ophthalmic clinicians and researchers seeking to appraise or conduct MR studies. Finally, we discuss future directions and emerging opportunities in the field, including multi-omic integration, expansion of ocular-specific datasets, advances in analytical methods, and the need to improve ancestral diversity to enhance the robustness and clinical relevance of MR findings.

## Introduction and background

Understanding causal relationships – whether factor A directly influences factor B – is a central challenge in epidemiology. While observational studies may demonstrate an association between two variables, this does not necessarily mean that the two are causally related (Figure 1). This is particularly important in biomedical research because only when a true causal relationship exists, will modifying an exposure (through a behavioural or therapeutic intervention, for example) result in a meaningful health outcome. Traditionally, researchers have relied on randomised controlled trials to establish causality; however, this study design is not always feasible or ethical, especially when considering long-term behaviours, environmental exposures, or potential harms. In this context, genetics offers a promising alternative to investigate causal relationships. By virtue of the fact that our genes are inherited randomly through the generations, we have all been recruited into many ongoing natural randomised controlled trials.

**Figure 1.**
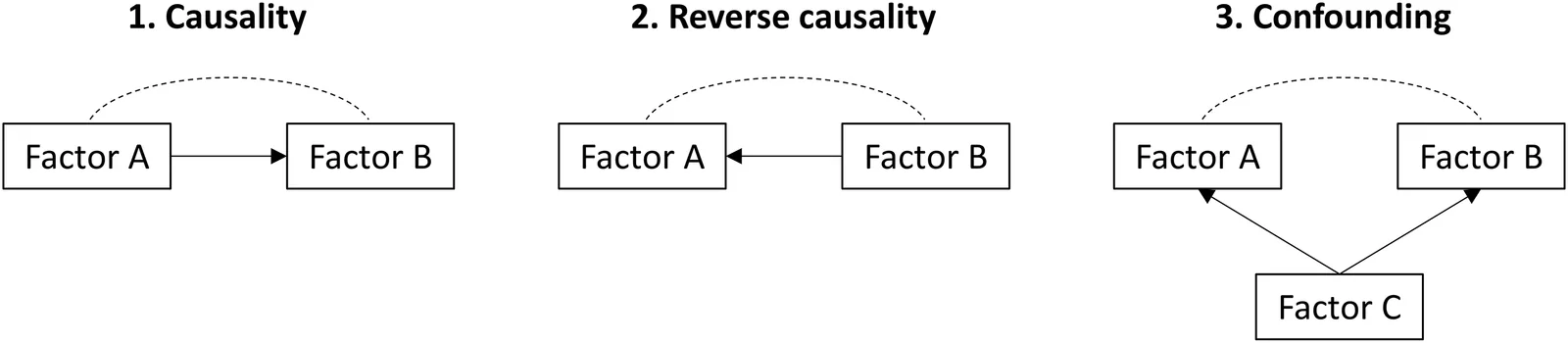
Conceptual models of causal and non-causal relationships.

Directed acyclic graphs illustrating the concepts of causality, reverse causality, and confounding. In each scenario, there is an observed association (dotted line) between factor A and factor B. This association may be the result of, (1) causality – factor A causes factor B (e.g., higher C-reactive protein levels cause ischaemic heart disease), (2) reverse causality – factor B causes factor A (e.g., ischaemic heart disease leads to higher C-reactive protein levels), or (3) confounding – a third factor (factor C) influences both factor A and factor B (e.g., older age is a risk factor for ischaemic heart disease and also results in higher C-reactive protein levels).

Mendelian randomisation (MR) is an epidemiological method that uses genetic variation to strengthen causal inference regarding the effect of modifiable exposures on health outcomes.^1^ The central premise is that genetic variants (termed instruments) robustly associated with an exposure of interest – rather than the exposure itself – are examined in relation to a particular outcome.^2^ A consistent association between such variants and the outcome provides evidence supportive of a causal relationship between the exposure and the outcome in question.

To illustrate, a notable example of this approach involves a functional variant (C677T) in the methylenetetrahydrofolate reductase (*MTHFR*) gene, which results in elevated homocysteine levels. If individuals carrying this variant also exhibit higher rates of a particular outcome, such as cardiovascular disease (CVD), this supports a potential causal role of homocysteine in CVD aetiology. Importantly, because genetic variants are fixed at conception and are generally independent of environmental and behavioural confounders, MR analyses are less susceptible to reverse causation and confounding than traditional observational studies (Figure 2).

**Figure 2.**
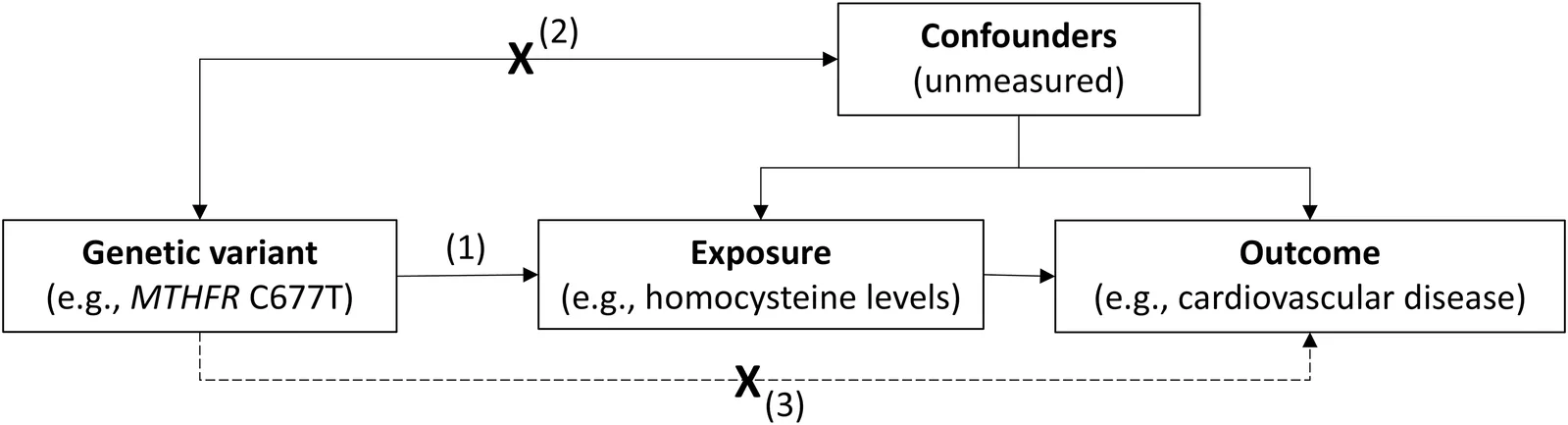
Principles and assumptions of Mendelian randomisation.

In this example, a functional genetic variant (C677T) in the methylenetetrahydrofolate reductase (*MTHFR*) gene, which is known to cause elevated homocysteine levels, is examined in relation to cardiovascular disease (CVD). If carriers of this variant exhibit higher rates of CVD than non-carriers, a causal relationship between homocysteine levels and CVD can be inferred. This inference is only valid when the following key assumptions are satisfied: (1) the genetic variant must be associated with the risk factor of interest (relevance assumption); (2) there are no unmeasured confounders of the gene-outcome association (independence assumption) (e.g., ancestral background may influence the relative frequency of genetic variants in the population and may also relate to the outcome of interest, introducing a potentially spurious association in a diverse cohort); and (3) the genetic variant affects the outcome only through the risk factor of interest (exclusion restriction assumption) (e.g., the *MTHFR* variant should not influence CVD through an association with body mass index, smoking, or other established CVD risk factors).

In this context, the *MTHFR* variant can be interpreted as a randomly allocated proxy for a low-folate diet (which is known to raise homocysteine levels), analogous to the allocation process in a randomised controlled trial (Figure 3). This framework has been applied to study the role of folate status and homocysteine levels across a range of outcomes – including neural tube defects,^3^ CVD,^4,5^ and glaucoma^6^ – often corroborating findings from observational and interventional research.^7–10^

**Figure 3.**
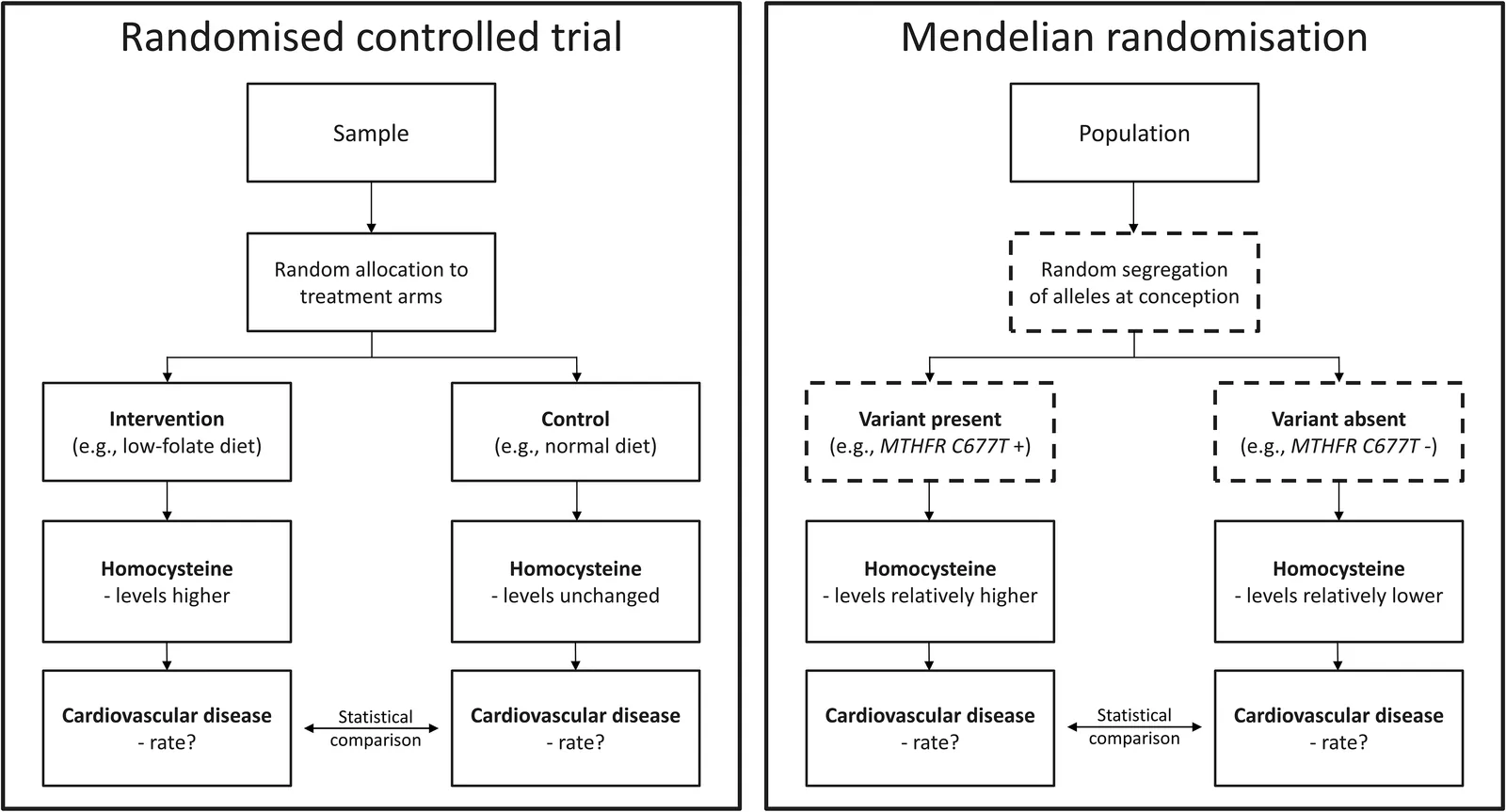
Mendelian randomisation as a natural randomised controlled trial.

Using the example of methylenetetrahydrofolate reductase (*MTHFR*) genetic variants, the random segregation of alleles at conception creates two separate populations groups, analogous to the random allocation of an intervention (e.g., a low-folate diet) in a randomised controlled trial. Confounders are assumed to be evenly distributed between the two groups with the only difference being the exposure of interest (in this case, homocysteine levels). If there are significant differences in the rates of the outcome of interest (e.g., cardiovascular disease), a causal relationship between the exposure and outcome can be inferred. Dashed boxes indicate the steps that occur by virtue of genetic inheritance rather than experimental intervention.

Early MR studies were instrumental in offering new insights into the causal effects of various circulating biomarkers – including inflammatory mediators, cholesterol fractions, and vitamins – predominantly in relation to CVD.^11–16^ These studies were characterised by a strong biological underpinning, with the chosen variants located in genomic regions directly related to the biomarker of interest (termed *cis*-MR). This approach can be particularly persuasive as the core MR assumptions (Figure 2) can be supported by prior understanding of genetic function.^2^

However, for complex traits (e.g., height or blood pressure), genetic variants from a single or handful of loci are unlikely to capture the complexity of the phenotype. With recent advances and improved access to genotyping technology, large genome-wide association studies (GWAS) have identified several hundreds, and sometimes thousands, of genetic variants associated with these, and other, traits.^17,18^ In response, statistical methods have been developed that can utilise multiple genetic instruments from across the genome as proxies for an exposure (termed genome-wide MR) through a variety of aggregate approaches or regression-based methods.^2^

Recent MR studies have increasingly used these methods to examine the putative causal effects of complex environmental, and even behavioural, traits on outcomes ranging from CVD to intraocular pressure (IOP) and myopia.^19–21^ However, since variants are chosen on a statistical rather than a biological basis, these MR studies have less of a mechanistic grounding and are generally more prone to violations of the core assumptions underlying valid causal inference.^2^ This underscores the importance of cautious interpretation and consideration of complementary lines of evidence, especially given the increased adoption of this technique across ophthalmic research.^22^

## Mendelian randomisation in ophthalmology

MR has been applied across a range of ophthalmic traits and conditions, using both genome-wide and *cis*-MR approaches. A particular strength of the method is its ability to address questions that would be otherwise impractical or unethical to study by traditional randomised controlled trials, such as the effects of smoking or educational attainment, or the causal role of potentially modifiable biomarkers.

Genome-wide MR studies have provided genetic evidence supporting a causal role of smoking on advanced age-related macular degeneration (AMD), as well as the relationship between pigmentation (measured through retinal fundus photographs) and skin cancer, reinforcing existing observational data and public health messaging.^23,24^ They have also confirmed well-established clinical risk factors for glaucoma, including higher IOP for primary open-angle glaucoma (POAG) and shorter axial length for primary angle-closure glaucoma (PACG), providing complementary lines of evidence to further support causal relationships.^25,26^ Bidirectional MR has clarified the direction of causality between educational attainment and myopia, suggesting that more time spent in education is itself a causal risk factor for myopic refractive error rather than myopia driving longer schooling.^21^ MR has additionally been used to investigate the role of myopia on retinal detachment, the relationship between behavioural and clinical factors with cataract, and the effect of complement proteins and lipid fractions on AMD.^27–30^

*Cis*-MR, which restricts genetic instruments to variants within or near a specific gene, can be used to model the effect of altering the level or function of the protein that gene encodes. The genetic variants for this are typically drawn from protein quantitative trait loci (pQTLs, variants associated with circulating protein levels) or expression quantitative trait loci (eQTLs, variants associated with gene expression levels in a given tissue). Because many such proteins are the targets of existing or potential drugs, *cis*-MR provides a genetic proxy for pharmacological intervention, offering a fast and inexpensive way to prioritise promising candidates for further evaluation in costly clinical trials.^31^ They may also be used to identify opportunities for drug repurposing or indication expansion, and to anticipate potential side effects of perturbing a given biochemical target.

For example, a *cis*-MR study of cholesteryl ester transfer protein (CETP) showed that CETP inhibition is likely to reduce the risk of coronary heart disease, while simultaneously flagging a potential increase in the risk of AMD as a possible adverse effect.^32^ It has also provided novel human genetic support for a causal role of TIE1 and TEK signalling in Schlemm's canal endothelium in lowering IOP, identifying both as potential therapeutic targets for glaucoma.^33^ Large multi-trait GWAS analyses combined with MR have further prioritised novel drug targets for POAG, including several that appear to act via optic nerve-related pathways rather than IOP.^34^

## Challenges and limitations

As evidenced above, MR has become an increasingly prominent approach in ophthalmic research, offering a framework to strengthen causal inference in the absence of feasible randomised controlled trials. In recent years, the scientific literature has witnessed an explosion of MR studies, driven largely by the widespread availability of publicly accessible GWAS summary statistics, the relative ease of implementing MR designs, and the emergence of automated and artificial intelligence-assisted analytical pipelines (Figure 4). While this democratisation of MR has accelerated output, it has introduced several key challenges that complicate the interpretation and reliability of findings.^35,36^

**Figure 4.**
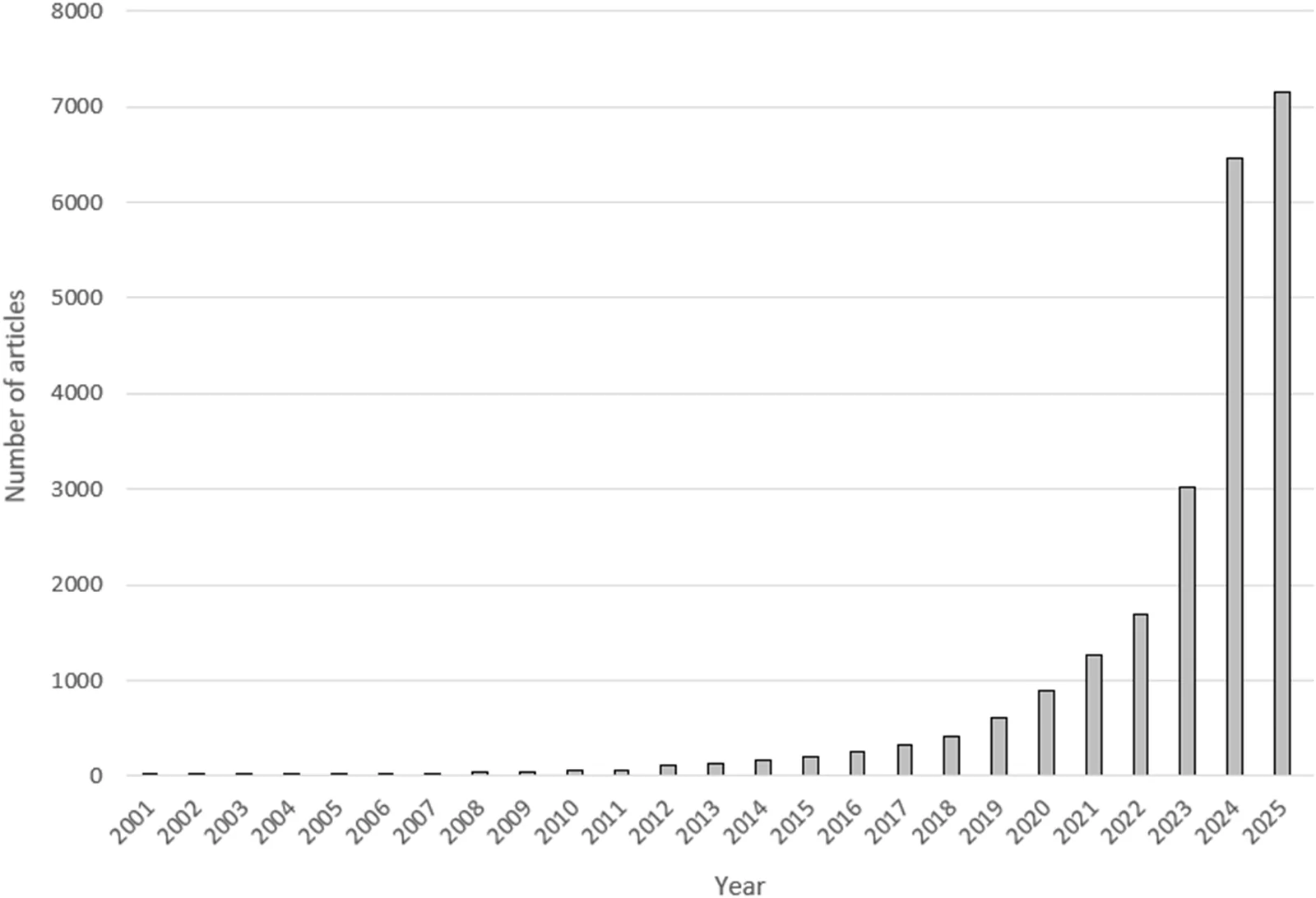
Published Mendelian randomisation studies over the last 25 years.

Graph depicts the number of PubMed entries with the terms “Mendelian randomisation” or “Mendelian randomization” in the title and/or abstract published annually from 2001 to 2025 (search conducted on 14 April 2026).

A particularly important, and often underappreciated, limitation is the assumption of gene-environment equivalence.^37^ Genetic proxies for exposures do not necessarily recapitulate the biological or temporal effects of modifiable environmental or behavioural factors. For example, genetic variants associated with educational attainment, smoking behaviours, or adiposity may capture lifelong predisposition to these traits rather than modifiable exposures at clinically relevant time points. This disconnect is especially problematic when interpreting MR findings for complex traits, where environmental, behavioural, and socioeconomic factors play substantial roles. Despite demonstrable statistical associations, the notion of there being specific genetic influences on many of the exposures examined in contemporary MR studies is implausible.^36^ As a result, causal estimates may simply reflect heritable confounding and are unlikely to translate into actionable clinical or public health interventions.^38^

Another important limitation relates to the core MR assumptions (demonstrated in Figure 2). In practice, these assumptions are difficult or impossible to verify and are often violated. Horizontal pleiotropy – where genetic variants influence multiple biological pathways – remains a particular concern. Although sensitivity analyses have been developed to detect and mitigate pleiotropy, these methods rely on additional assumptions and cannot fully eliminate bias.^2^ The widespread adoption of two-sample MR – where gene-exposure and gene-outcome associations are measured in separate cohorts – has further amplified these challenges. By leveraging publicly available GWAS datasets, researchers can rapidly test numerous hypotheses without access to individual-level data. High-throughput analyses can generate large numbers of statistically significant associations, but without careful consideration of biological plausibility and methodological limitations, these findings risk being misleading.^35,36^

Another important limitation is the interpretability of MR point estimates.^39^ Fundamentally, MR tests the null hypothesis that there is no difference in outcomes between the genotypes associated with higher or lower levels of the risk factor of interest. Such causal null hypothesis testing requires fewer assumptions, centred on those core assumptions of instrument validity described above, and can help establish the presence and direction of a causal effect of the exposure on the outcome.^40^ Scaling the genetic effect into a well-defined exposure effect estimate requires further parametric assumptions, which may not be plausible, and thus MR point estimates should not be interpreted literally. Of course, a well-defined but biased estimate of an effect size is of little use and so the challenges of interpreting MR estimates must be weighed against the genuine value of the approach in reducing bias.

Finally, it is imperative that results from MR studies be triangulated with other lines of evidence, including traditional observational epidemiology, experimental studies, and where possible, clinical trials.^41^ MR should not be viewed in isolation but rather as one component of a broader causal inference framework. A lack of concordance between MR findings and observational data should prompt scrutiny rather than immediate dismissal of one approach.

## Practical considerations

Vision researchers reading or undertaking MR analyses are encouraged to consult established methodological guidance on the design, interpretation, and reporting of such studies.^42,43^ A summary of important considerations, including reassuring features and common shortcomings of MR studies, is presented in Table 1. While there is no single standard approach to MR studies, and not all items may apply in every case, this checklist may provide a simple tool for clinicians to gauge the reliability and validity of reported MR studies. Accessible introductory resources are also available which provide a practical glossary and checklist tailored for clinicians new to the approach.^2^ A robust MR study should incorporate four key elements: (1) appropriate data selection and instrument choice, (2) sensitivity analyses to interrogate core assumptions, (3) cautious and context-aware interpretation of findings, and (4) adherence to recognised reporting standards.^44^

**Table 1.** Appraisal tool for clinicians interpreting or conducting Mendelian randomisation studies.

Appraisal criterion	Green flags (reassuring factors)	Red flags (exercise caution)
Instrument selection and grounding	Uses *cis*-MR or instruments mapping directly to target genes where the biological and functional mechanism is well-characterised.	Exclusive reliance on genome-wide instruments selected purely via statistical thresholds without known functional or biological pathways.
Gene-environment equivalence	Clear evidence or strong rationale that the lifelong genetic proxy accurately models the physiological mechanism or downstream effects of the proposed intervention.	Implausible genetic proxies for complex behavioural or socioeconomic traits (e.g., assuming a genetic instrument perfectly mimics behavioural choices in adulthood).
Sample overlap	Uses independent, non-overlapping cohorts for the exposure data and the outcome data in a two-sample MR design.	High degree of participant overlap between the exposure and outcome datasets in the presence of weak instruments, inflating the risk of false-positive findings.
Ancestry alignment	The ancestral backgrounds of the exposure and outcome cohorts are explicitly matched (e.g., both cohorts are of the same distinct ancestry).	Mixing disparate ancestral data, which introduces confounding due to differences in linkage disequilibrium and allele frequencies across populations.
Reverse causality testing	Utilizes formal bidirectional MR and specific filtering techniques (such as Steiger filtering) to increase the likelihood that the genetic variant influences the exposure prior to affecting the outcome.	Assumes a unidirectional pathway without testing; ignores the possibility that early, subclinical manifestations of the outcome are driving changes in the exposure.
Multivariable adjustment	Employs multivariable MR when dealing with closely related or highly correlated exposures to isolate the independent causal effect of interest.	Fails to account for genetically correlated exposures, leaving open the possibility that the observed effect is driven by pleiotropy.
Statistical diagnostic reporting	Explicitly reports tests for directional pleiotropy (e.g., MR-Egger intercept) and quantifies instrument heterogeneity (e.g., Cochran's Q or I^2^ statistics).	Omits diagnostic testing for pleiotropy or heterogeneity, presenting only the primary causal p-values.
Sensitivity analyses	Employs multiple pleiotropy-robust statistical methods (e.g., MR-Egger, weighted median, mode-based estimators) and tests for violations like horizontal pleiotropy.	Reports only a standard inverse-variance weighted (IVW) analysis, which is sensitive to outliers, with missing sensitivity testing.
Evidence triangulation	Explicitly cross-references and integrates findings with traditional epidemiology, animal models, or existing clinical data.	Views MR in isolation; presents the genetic data as definitive, standalone proof of causality, or ignores conflicts with large observational trials.
Interpretation of null findings	Interprets a non-significant result cautiously, mindful that a null result may reflect limited statistical power.	Explicitly states that a non-significant p-value definitively proves a lack of causal effect, without evaluating statistical power or biological compensation.
Interpretation of effect sizes	Focuses on the presence and direction of a causal effect; acknowledges that point estimates represent a lifelong exposure and cannot be interpreted literally as an acute trial effect.	Overinterprets the exact point estimate or effect size as a direct prediction of what a clinical trial or treatment will achieve.

The initial step involves precise formulation of the research question and identification of suitable data sources for the genetic instruments, exposure, and outcome. These data may comprise individual-level or summary statistics and may be derived from a single cohort (one-sample MR) or from two independent samples (two-sample MR). Instrument selection may be restricted to a single genomic locus (*cis*-MR) or extend across multiple loci (genome-wide MR).

Irrespective of the chosen design, it is essential to evaluate whether a biologically plausible and empirically supported pathway links the genetic instrument to the exposure. This is particularly relevant to the principle of gene-environment equivalence, where the effect of a genetic proxy is assumed to be mechanistically similar to that of a modifiable exposure or therapeutic intervention. This concept is central to MR and lack of equivalence can negate any causal inference from the outset.^37^ In *cis*-MR, where variants are located within or proximal to the gene encoding the exposure of interest, this assumption is often supported by prior functional evidence. In contrast, for genome-wide MR, the underlying biological mechanisms are frequently less well characterised, increasing susceptibility to violations such as horizontal pleiotropy.

As outlined above, valid causal inference in MR rests on three core instrumental variable assumptions: relevance, independence, and exclusion restriction. In practice, each assumption is susceptible to specific violations and appropriate sensitivity analyses should be undertaken to probe these. For example, canalisation – the ability of a biological system to maintain a stable phenotype through compensatory mechanisms despite underlying genetic variation – may weaken instrument relevance; population stratification can influence independence if allele frequencies differ across ancestral groups with distinct disease risks; and horizontal pleiotropy may violate the exclusion restriction assumption by introducing alternative pathways between the genetic instrument and the outcome (Figure 2). A variety of sensitivity and supplementary analyses can be employed to probe for potential violations of these assumptions and to gauge the robustness of results.^2,42^ These include testing associations of genetic instruments with directly measured exposures or potential confounders, the use of pleiotropy-robust statistical methods, and comparison with appropriate positive or negative controls. Additional considerations include bidirectional, multivariable, colocalisation, and subgroup analyses, depending on the specific research question at hand.

Completed analyses should be reported in accordance with the STROBE-MR guidelines, which provide a structured framework for transparent reporting of instrument selection, assumption justification, and sensitivity analyses.^43^

Importantly, MR should be interpreted within the broader context of causal inference and not as a standalone source of evidence. While MR can strengthen support for a causal relationship, it cannot establish causality definitively. Findings should therefore be triangulated with evidence from observational studies, experimental research, and, where feasible, randomised controlled trials.^41^ Comparisons between MR and observational estimates, including formal tests for heterogeneity, may provide additional insight into potential sources of discrepancy.

Furthermore, MR estimates typically reflect the effect of lifelong differences in exposure. As a result, effect sizes may differ from those observed in interventional studies, where the timing, magnitude, and duration of the intervention are different. Estimates may be inflated relative to randomised trials or attenuated due to compensatory biological processes such as canalisation. Null findings should also be interpreted cautiously. Absence of evidence for a causal effect may reflect limited statistical power, weak instruments, or violations of underlying assumptions rather than a true null relationship.

In summary, for ophthalmologists appraising the MR literature, studies that incorporate biologically informed instrument selection, transparent and comprehensive sensitivity analyses, and corroboration across independent datasets are likely to provide the most reliable and clinically informative evidence.

## Future directions, developments, and opportunities

A particularly promising direction for MR is the scaling up of *cis*-MR from single-gene, hypothesis-driven analyses to high-throughput, proteome-wide screens. This has been made possible by the rapid expansion of large-scale plasma pQTL datasets such as the UK Biobank Pharma Proteomics Project and deCODE genetics, which together characterise the genetic determinants of thousands of circulating proteins.^45,46^ These resources enable hypothesis-free screens of all potentially druggable proteins against an ocular disease outcome in a single analysis.^34,47^

Equally important is the continued expansion of GWAS for ocular traits and conditions themselves, which supply the outcome data for MR analyses. Larger sample sizes improve statistical power and enable more meaningful subgroup analyses, for example by disease subtype. Stratified analyses of this kind can reveal biology that is obscured when subtypes are pooled: the International AMD Genomics Consortium GWAS, for example, identified the first genetic signal specific to neovascular AMD near *MMP9*, a finding that would not have emerged from analysis of advanced AMD as a single phenotype.^48^

A significant limitation of current *cis*-MR approaches is the reliance on blood-based proteomic data. Because the pQTL datasets used in *cis*-MR measure circulating plasma proteins, the utility of blood pQTLs for ocular disease varies by condition and may be limited where the protein of interest is not well represented in the circulation.^31^ Tissue-specific ocular QTL resources exist but remain modest in scale: the EyeGEx dataset provides retinal eQTLs from approximately 450 postmortem donor retinas, and a smaller retinal eQTL mega-analysis has been conducted in approximately 311 samples.^49,50^ Neither approaches the scale of blood-based resources, and GWAS-scale proteomic QTL data from ocular tissues or fluids such as aqueous humour or vitreous do not yet exist. Increasing the scale and tissue diversity of ocular molecular datasets is therefore an important priority for the field.

The credibility of causal inferences can be strengthened by integrating evidence across multiple omics layers.^41^ Concordance between a *cis*-MR signal using pQTLs, colocalisation with an eQTL in the relevant tissue, and supportive associations with downstream metabolites or imaging-derived ocular biomarkers provides a far more compelling basis for drug target prioritisation or repurposing than any single line of evidence in isolation. Early examples of this integrated approach in ophthalmology combine proteome-wide MR with colocalisation and proteome-wide association studies for glaucoma drug target discovery.^34,47^

Finally, the generalisability of findings from MR in ophthalmology is currently limited by the predominantly European ancestry of the underlying GWAS data. Given substantial differences in allele frequencies, linkage disequilibrium structure, and disease prevalence across ancestries – for example, the higher burden of POAG in African-ancestry populations and of myopia in East Asian populations – expanding the diversity of both exposure and outcome GWAS is essential to ensure that MR-derived insights translate equitably across the populations most affected.^51^ large-scale initiatives such as the Human Heredity and Health in Africa (H3Africa) consortium, and cross-ancestry analyses within ocular GWAS, represent important steps in this direction.^34,52^

## Conclusion

MR has become an increasingly influential tool in ophthalmic research, offering a means to strengthen causal inference from observational data, especially where conventional experimental approaches are impractical or unethical. Its application has provided valuable insights into disease mechanisms, risk factors, and potential therapeutic targets, particularly with the integration of large-scale genomic and proteomic datasets. However, the validity of MR findings depends on critical underlying assumptions, the biological plausibility of selected instruments, and the robustness of accompanying sensitivity analyses.

As methodological complexity and data availability continue to expand, there is a corresponding need for careful interpretation and rigorous study design.^35,36^ MR should therefore be viewed not as definitive evidence of causality, but as one component of a broader evidential framework. When applied appropriately, transparently, and interpreted alongside complementary lines of evidence, MR can contribute meaningfully to advancing understanding and guiding clinical translation in ophthalmology.

## Financial support

**KVS**: Fight for Sight (1956A), The Desmond Foundation, National Institute for Health and Care Research (NIHR) Moorfields Biomedical Research Centre (BRC) Career Enhancement Award. **ANW**: Wellcome Trust (224390/Z/21/Z). **CLP**: Fight for Sight (RESZAK2402). **SR**: Moorfields Eye Charity Springboard Award (SB-25B-104). **APK**: UK Research and Innovation (UKRI) Future Leaders Fellowship (MR/T040912/1), Moorfields Eye Charity Career Development Fellowship, Lister Institute of Preventative Medicine Fellowship. **KVS**, **ANW**, **CLP**, **SR**, **APK**: Supported by a grant from the National Institute for Health and Care Research (NIHR) for a Biomedical Research Centre (BRC) at Moorfields Eye Hospital NHS Foundation Trust and UCL (University College London) Institute of Ophthalmology.

The sponsors or funding organisations had no role in the design or conduct of this research.

For the purpose of open access, the author has applied a Creative Commons Attribution (CC BY) license to any Author Accepted Manuscript version arising.
